# Male occult breast cancer with features highly resembling primary lung cancer: a case report and literature review

**DOI:** 10.3389/fonc.2025.1737310

**Published:** 2026-01-12

**Authors:** Hao Su, Xinyu Zhao, Huayu Liu, Huantong Shu, Wenjing Li, Zhimin Yang, Mianli Li

**Affiliations:** Department of Oncology, Binzhou Medical University Hospital, Binzhou, Shandong, China

**Keywords:** case report, male breast cancer, occult breast cancer, primary lung cancer, systemic therapy

## Abstract

Male breast cancer is an extremely rare form of malignant tumor, and occult breast cancer is also an exceptionally uncommon disease. Given its rarity, diagnosing occult breast cancer in male patients presents significant challenges, and the diagnosis becomes even more difficult when the tumor exhibits characteristics of other neoplasms. For instance, this case report describes a male patient with occult breast cancer presenting with features typical of primary lung cancer. The uniqueness and complexity of this case lie in its clinical presentation: beyond typical axillary lymph node enlargement, the primary imaging feature was an isolated pulmonary mass lesion. This presented significant diagnostic hurdles. For the diagnosis of this case, we administered chemotherapy and intracranial radiotherapy to the patient. Therefore, sharing this rare case aims to heighten clinicians’ awareness of differential diagnoses for metastatic cancer with an unknown primary site, particularly when pathological findings of pulmonary lesions do not align with conventional lung cancer markers.

## 1 Introduction

Male breast cancer is a rare malignant tumor, and occult breast cancer is also an uncommon type of breast cancer characterized by presenting primarily with axillary lymph node or distant organ metastases. Today we discuss a case of occult breast cancer in a male patient exhibiting highly characteristic features of primary lung cancer. The unique aspect of this case lies in its clinical presentation: beyond the typical feature of axillary lymph node enlargement, the most significant manifestation was an isolated pulmonary mass lesion. Its imaging features are highly characteristic of primary lung cancer, presenting significant diagnostic and therapeutic challenges.

## 2 Case report

This case involves a 55-year-old male patient with no family history of breast cancer or other tumors. He has no history of long-term hormone medication use or other factors known to induce breast cancer. There is also no evidence of exposure to epidemiological risk factors. Furthermore, there is no significant past history indicating the presence of a hereditary disease.

**Figure 1:**
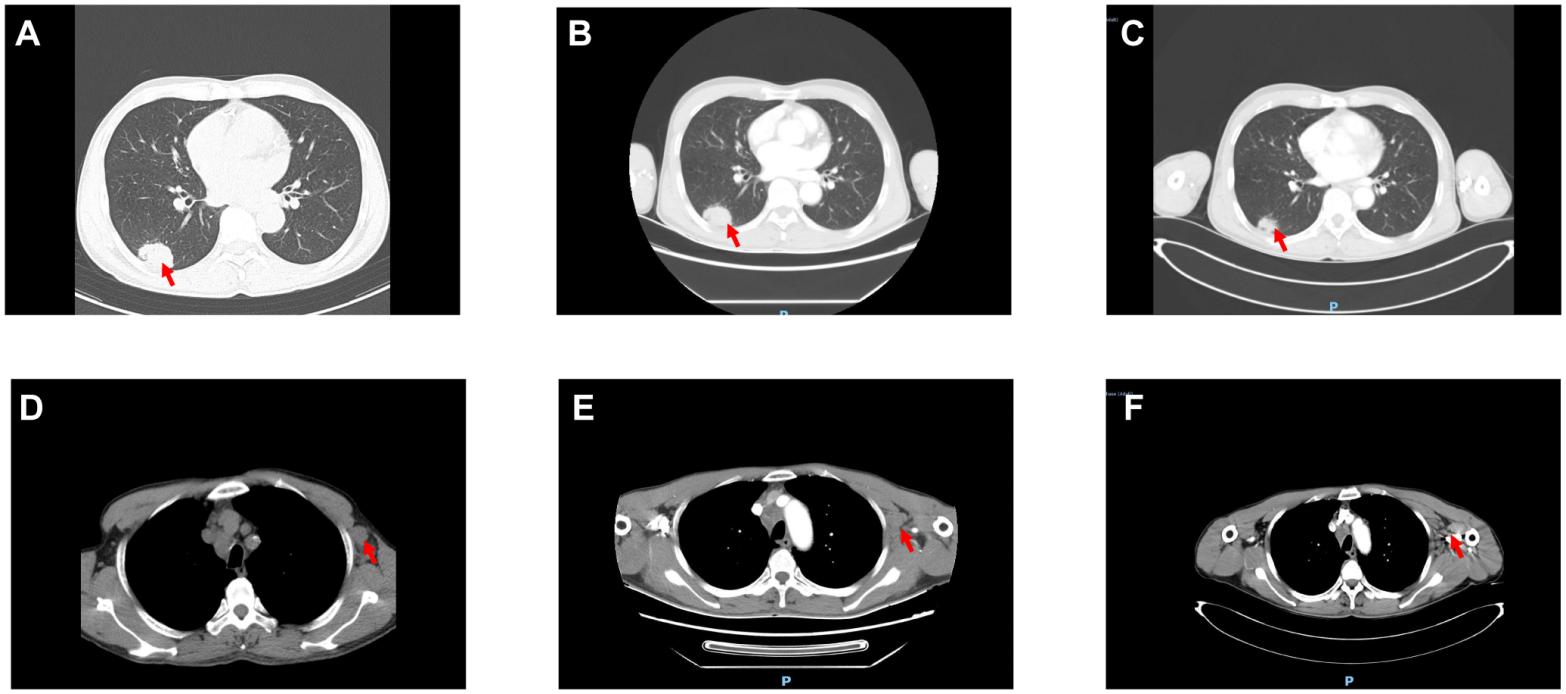
Changes in the pulmonary mass and left axillary lymph nodes. **(A-F)** Shows the right lung lessions and left axillary lymph node lesions on June 1, June 4, and July 21, 2025, respectively. (The red arrows point to the lesions).

The patient first presented to a local hospital on June 1, 2025, complaining of cough, sputum production, and chest tightness. A chest CT scan revealed an approximately 3.7 × 2.5 cm space-occupying lesion in the right lower lobe (**Figure 1A**), accompanied by enlarged left axillary lymph nodes (**Figure 1D**). Given the patient’s symptoms of coughing, expectoration, and chest tightness—similar to pneumonia—and considering the limitations of lower-tier hospitals, the local hospital suspected a pulmonary infection and administered symptomatic treatment with budesonide, salbutamol, and moxifloxacin.

**Figure 2:**
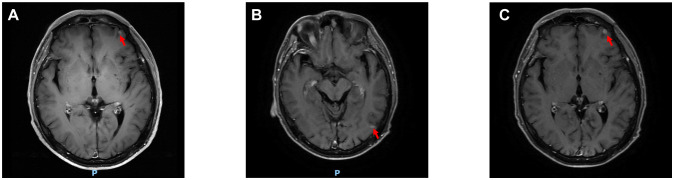
Changes in cranial lesions. **(A-C)** Shows the cranial lesions on June 4, July 21, and September 3, 2025, respectively. (The red arrows point to the lesions).

**Figure 3:**
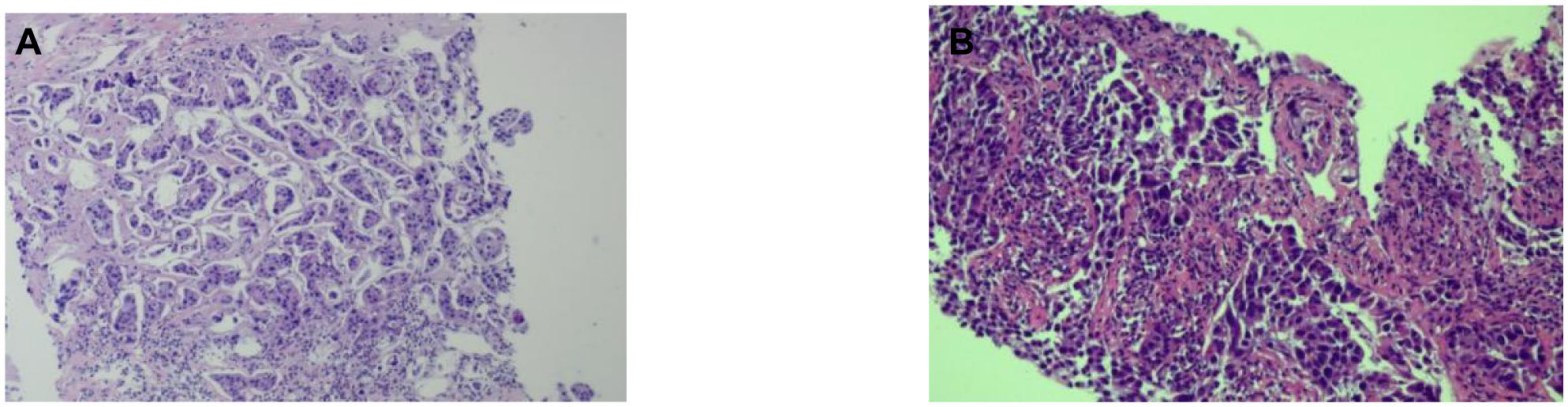
HE-stained results of axillary lymph node and pulmonary mass biopsy. **(A)** HE staining results of the left axillary lymph node biopsy. **(B)** HE staining results of the right lung lesion biopsy.

**Figure 4:**
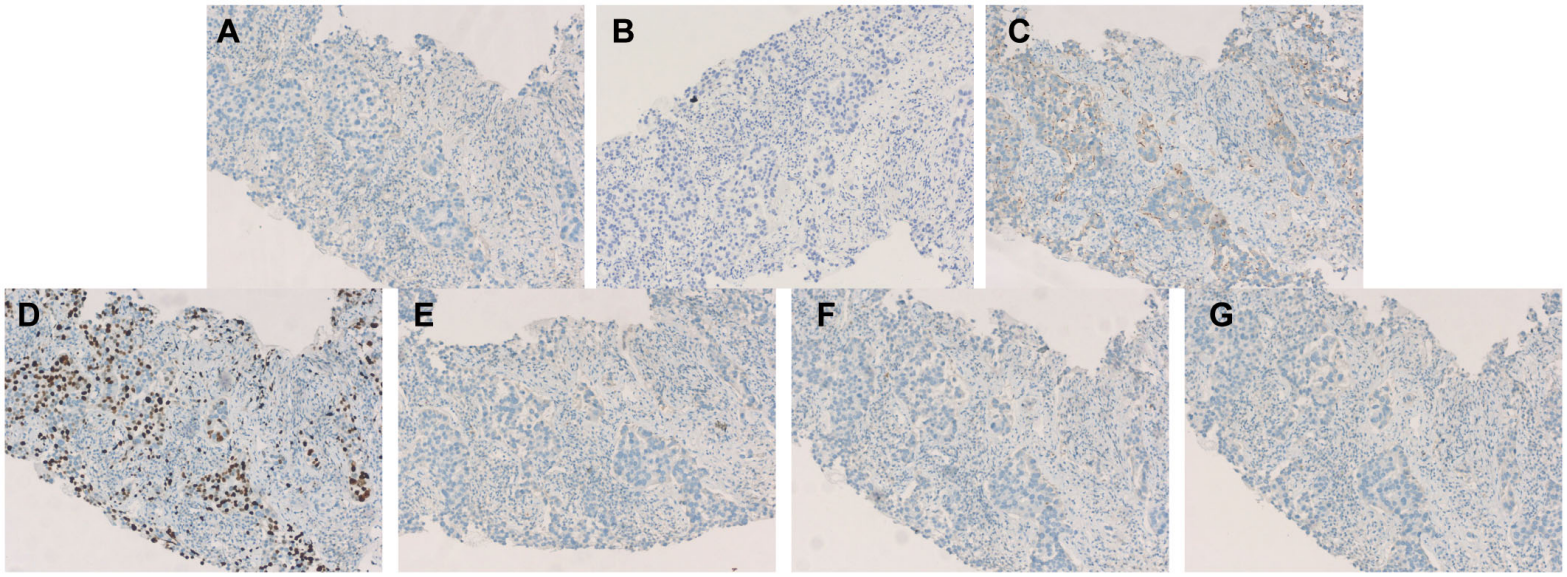
Immunohistochemistry **(A)** Immunohistochemistry for ER: negative. **(B)** Immunohistochemistry for PR: negative. **(C)** Immunohistochemistry for Her-2: negative (1+). **(D)** Immunohistochemistry for Ki-67 ≈70%. **(E)** Immunohistochemistry for Mammaglobin: negative. **(F)** Immunohistochemistry for TTF-1:negative. **(G)** Immunohistochemistry for Napsin A: negative.

Due to poor response to symptomatic treatment, the patient presented to Binzhou Medical University Affiliated Hospital on June 4, 2025. Local imaging studies and a comprehensive physical examination revealed palpable enlarged lymph nodes in the left axilla. Further testing showed carcinoembryonic antigen (CEA): 28.32 ng/ml, and CY21-1 (non-small cell carcinoma marker): 47.68 ng/ml. This temporarily ruled out pulmonary infection, raising suspicion of a pulmonary tumor. Enhanced CT of the neck, chest, and upper abdomen revealed an occupying lesion in the right lower lobe measuring approximately 36 mm × 23 mm (**Figure 1B**), accompanied by enlarged lymph nodes in the left axilla (**Figure 1E**). Further evaluation with contrast-enhanced cranial MRI and DWI showed a nodule in the left frontal lobe (**Figure 2A**). Based on the patient’s history, this is considered metastatic tumor. After excluding contraindications, an ultrasound-guided core needle biopsy of the left axillary lymph node was performed on June 6, 2025. The pathology report dated June 11, 2025, stated: (Axillary lymph node) Adenocarcinoma was identified within the fibrous connective tissue of the biopsy specimen, showing a partially micropapillary pattern. Based on morphology and immunohistochemical staining, it is considered to originate from the breast. Further investigation is recommended. Immunohistochemistry: estrogen receptor (ER) (+), progesterone receptor (PR) (-), GATA-binding protein-3 (GATA-3) (+), gross cystic disease fluid protein-15 (GCDFP-15) (–), trichorhinophalangeal syndrome type1 (TRPS1) (+), cytokeratin 7 (CK7) (+), Napsin A (–), thyroid transcription factor-1 (TTF-1) (–), cytokeratin 20 (CK20) (–), Villin (–), P40 (–), human epidermal growth factor receptor-2 (Her-2) (2+, FISH-), Ki-67 proliferation index approximately 80% (**Figure 3A**). Further refinement of breast and axillary lymph node ultrasound: 1. Bilateral retromammary patchy hypoechoic areas suggest possible male breast development; space-occupying lesion to be excluded (**Supplementary Figure S1**). 2. Low-echo lesion in the left axilla (**Supplementary Figure S2**). Further examination of carbohydrate antigen 153 (CA153): 541.20 U/mL, carbohydrate antigen 125 (CA125): 104.00 U/mL. At that time, the possibility of occult breast cancer in this patient had not yet been considered. Consequently, a CT-guided right lung mass biopsy was performed on June 12, 2025. Pathology findings from the right lung mass biopsy: (Right lung) Poorly differentiated adenocarcinoma. Based on the medical history and immunohistochemistry, a breast origin cannot be excluded. Immunohistochemistry: GATA-3 (+ in scattered cells), TRPS1 (+), Mammaglobin (–), ER (0), PR (0), Her-2 (1+, low expression), TTF-1 (–), (**Figure 3B**) Napsin A (–), P40 (–), CK7 (–), Ki-67 (≈70%) (**Figures 3B**, **4**). Based on the patient’s tumor marker levels, imaging studies, and pathological examination results, the patient was diagnosed with stage IV occult breast cancer with metastases to the lungs and brain. Due to the patient’s resistance to chemotherapy, the TP regimen was not selected. Instead, the shorter-duration TX regimen was chosen: docetaxel (DOC) 130mg on Day 1 + capecitabine (CAP) 1.5g bid on Days 1-14 (1 cycle). This was followed by endocrine therapy. Further imaging revealed: enlarged left axillary lymph nodes. The discharge diagnosis was poorly differentiated breast adenocarcinoma, stage IV (cTxN3M1).

The patient was readmitted on July 21, 2025, with updated tumor markers: CEA 32.6 ng/mL, CA125 131.0 U/mL, and CA153 900.2 U/mL, all elevated compared to previous levels. DOC was replaced with albumin-bound paclitaxel (nab-P) 400 mg on Day 1 (D1) plus CAP 1.5 g twice daily (bid) from D1 to D14. Subsequent treatment with this regimen continued for 3 cycles, during which tumor markers showed consistent decline (**Supplementary Figure S3**). Follow-up CEA on September 3, 2025: 20.8 ng/mL; CA125: 63.28 U/mL; CA153: 635.20 U/mL. Follow-up contrast-enhanced CT of the neck, chest, and upper abdomen: The soft tissue mass in the right lower lobe has decreased in size compared to previous imaging, now measuring approximately 25mm × 22mm (**Figure 1C**). The left axillary lymph node has decreased in size compared to previous imaging (**Figure 1F**). Follow-up cranial MRI with dynamic contrast enhancement and DWI: The pre-existing hyperintense lesion in the left frontal lobe has increased in size compared to previous imaging. New hyperintense lesions are noted in the left frontal lobe (**Figure 2B**) and the junction of the left temporal and occipital lobes (**Figure 2C**). Based on comprehensive imaging and laboratory findings, the intracranial lesion was assessed as progressive while the extracranial lesion showed improvement. Treatment was continued with one cycle of nab-P plus CAP. Intracranial radiotherapy was administered from September 22, 2025, to October 17, 2025, with the following specific dose: 4000 cGy/20f/4w. At the final follow-up on September 22, 2025, CEA was 18.61 ng/ml, CA125 was 52.85 U/ml, and CA153 was 631.60 U/ml.

## 3 Discussion

Male breast cancer is a rare malignant tumor. According to relevant literature, male breast cancer in China accounts for only 0.31% of the overall breast cancer population and 0.6% of the global breast cancer population (1). Public awareness of this disease remains relatively low, leading to most patients presenting at an advanced stage during their initial visit, which significantly impacts prognosis. Among all breast cancers, occult breast cancer accounts for only 0.1% to 0.8% of cases. Occult breast cancer is equally rare in male breast cancer, representing approximately 0.2% to 0.9% of cases (2). The hallmark feature of occult breast cancer is axillary lymph node metastasis. The absence of an identifiable primary tumor within the breast makes it a challenging entity to diagnose. In this case, beyond presenting with typical axillary lymph node metastasis at initial diagnosis, the patient also exhibited distant organ metastases, such as to the brain and lungs, further complicating our diagnostic and therapeutic approach. MRI demonstrates high sensitivity in diagnosing breast cancer, particularly in cases where mammography or ultrasound struggles to identify lesions. It can detect malignancies even when other modalities fail, achieving detection rates exceeding 90% for dense breasts and microcalcifications (≤5 mm). However, approximately 10% of patients still fail to show primary lesions on MRI, particularly when the lesions are extremely small or located deep within the breast tissue (3). As demonstrated in this case, the breast MRI findings still failed to suggest the possibility of breast cancer. this highlights the limitations of relying solely on imaging for diagnosis. Isolated pulmonary lesions cannot be definitively distinguished on imaging as either primary lung cancer or isolated metastatic lesions from breast cancer. Furthermore, although both breast ultrasound and breast MRI in this patient suggested gynecomastia, and the patient had no history of hormone medication use, this may offer some diagnostic clues for breast cancer. However, pathological examination remains the definitive step for establishing a diagnosis.

In terms of cancer histopathological characteristics, comparing males and females reveals that due to the absence of lobular structures in male breasts, invasive ductal carcinoma is more common in male patients (83.7% vs. 77.8%), while invasive lobular carcinoma is more prevalent in female patients (1.3% vs. 4.6%). This pattern aligns with histological statistics for male breast cancer patients in China. In recent years, invasive ductal carcinoma accounted for approximately 79.5% of cases among male patients, with the majority (69.8%) classified as moderately differentiated (Grade II) (1, 4, 5). Regarding the expression of ER and PR in cancer cells, the probability of both ER and PR being positive is significantly higher for both male and female patients than the probability of either ER or PR being positive alone or both being negative (6). Regarding HER-2 expression, male and female patients exhibit comparable rates of positivity. Regarding another key prognostic indicator—Ki-67—approximately 55% of both male and female patients exhibit Ki-67 levels exceeding 20%. Due to increased attention in recent years, the rate of missing Ki-67 data among male breast cancer patients in China has significantly decreased (46.1% before 2009 vs. 5.8% after 2020) (1, 7). Multiple multicenter studies indicate that both male and female patients predominantly present with Luminal A&B subtypes, with female patients exhibiting a higher proportion of triple-negative or HER-2-positive cases (5, 8). In addition, positive results for GCDFP and Mammaglobin are compelling indicators suggesting a mammary origin of the lesion (9). However, in this case, both GCDFP-15 and Mammaglobin were negative, complicating the definitive diagnosis. The commonly used biomarkers for diagnosing pulmonary tumors are TTF-1 and Napsin A. TTF-1 is positively expressed in the vast majority of lung adenocarcinomas, and Napsin A also exhibits high sensitivity and specificity (10). In this case, both TTF-1 and Napsin A immunohistochemical results were negative, strongly suggesting that the lesion did not originate in the lungs. Concurrently, GATA-3 is recognized as one of the most reliable markers for distinguishing breast cancer from lung adenocarcinoma (9). Its expression is highly prevalent in breast cancer but rare in lung adenocarcinoma. In this case, both rounds of GATA-3 immunohistochemistry yielded positive results. In this case, the positive expression of GATA3, ER, and PR in tumor cells, combined with negative results for TTF-1 and Napsin A, definitively indicates that the tumor originated in the breast. This combination of negative pulmonary markers and positive breast markers serves as the gold standard for determining whether a pulmonary mass originates from the breast, even when the primary breast lesion is occult.

The results of the pathological examination provide guidance for subsequent treatment. However, it is noteworthy that we obtained two separate pathological specimens from this patient—one from the axillary lymph node and another from the pulmonary mass—and immunohistochemical analysis revealed differing pathological outcomes: one specimen was classified as triple-negative, while the other was identified as Luminal B type. This phenomenon was previously concluded in the study by Weydandt et al.—such inconsistencies may arise as early as the initial stage of tumor metastasis to regional lymph nodes. Through simultaneous needle biopsies of the primary tumor core and axillary lymph node metastases in patients, they observed significant discrepancies in the expression of ER, PR, HER-2, and Ki-67 (11). Beyond tumor heterogeneity, technical factors may also contribute to inconsistent final results, such as variations in specimen processing, limitations of detection methods, and differences in pathologist interpretation (12). Although the phenomenon of biomarker inconsistency between primary tumors and metastatic sites is real, the probability of such inconsistency varies. For instance, Khedr et al. found that the incidence of ER and HER-2 conversion was relatively low. Their study also revealed that these changes in biomarker expression were associated with patient prognosis. For instance, patients in the HER-2 low-expression group demonstrated poorer outcomes when HER-2 expression at distant metastatic sites shifted to negative. Conversely, those with consistent ER and PR expression at both primary and metastatic sites exhibited better prognosis (13). Therefore, when faced with two different pathology reports for the same patient, selecting which pathology result to use as the basis for guiding subsequent treatment is critical. The pathology results for the patient’s axillary and pulmonary masses indicate that only the axillary lymph node fine-needle aspiration biopsy showed ER-positive status. Integrating the findings from these two distinct sites, we propose a diagnosis of triple-negative breast cancer for this patient. Given the ER-positive axillary lymph node result, we have decided to incorporate endocrine therapy into the subsequent treatment regimen.

Once the diagnosis was confirmed that the pulmonary mass originated from breast cancer, the treatment approach was completely altered. Had it been misdiagnosed as primary lung cancer, subsequent therapy would have been guided by testing for driver genes such as EGFR, ALK, or ROS1, leading to the selection of corresponding targeted therapies. However, these gene-targeted drugs are ineffective for breast cancer. Currently, treatment decisions for male breast cancer patients should align with those for female breast cancer patients (14). The Chinese Society of Clinical Oncology (CSCO) and the Chinese Anti-Cancer Association’s breast cancer diagnosis and treatment guidelines both list the TX regimen as the recommended chemotherapy regimen for stage III and stage IV advanced breast cancer. Other chemotherapy regimens for advanced breast cancer include TP, NX, etc (15). TP, as a more potent chemotherapy regimen, demonstrates superior survival outcomes compared to the TX regimen. For instance, in a study by Fan et al., 53 patients with advanced metastatic triple-negative breast cancer were randomized to receive either TP or TX for six cycles. Results revealed significant improvements in ORR, PFS, and OS in the TP group versus the TX group (63.0% vs. 15.4%, 10.9 months vs. 4.8 months, 32.8 months vs. 21.5 months) (16). In the TX regimen, DOC is the drug of choice. However, nab-P demonstrates superior antitumor efficacy compared to DOC. The CA024 study revealed that nab-P significantly improved the objective response rate (ORR) compared to DOC in treating patients with previously untreated metastatic breast cancer (74% vs. 39%, respectively), with extended median progression-free survival (mPFS) (14.6 vs. 7.8 months) and median overall survival (mOS) (33.8 vs. 26.6 months) (17). Therefore, if the patient does not resist chemotherapy and can tolerate platinum-based drugs, in accordance with NCCN and CSCO guidelines, we would prefer the TP regimen. Given this patient’s circumstances, we would have chosen it as well. However, the patient expressed reluctance toward chemotherapy. Consequently, for this patient with advanced triple-negative breast cancer, we selected the TX regimen. Following tumor marker elevation, docetaxel was promptly replaced with albumin-bound paclitaxel. After TX treatment, significant reduction was observed in the pulmonary metastases and left axillary lymph nodes.

Regarding endocrine therapy, considering the patient’s positive ER status in axillary lymph nodes, we recommend endocrine medication following chemotherapy to further control metastatic lesions and improve the patient’s prognosis. The American guidelines for the diagnosis and treatment of male breast cancer recommend that hormone receptor-positive male patients receive endocrine therapy with tamoxifen 20mg for 5 years. Even for patients who have completed 5 years of tamoxifen therapy but remain at high risk of recurrence, an additional 5 years of tamoxifen therapy is still advised (14). In a prospective study of 448 male breast cancer patients, half had positive axillary lymph nodes, and 98.4% were hormone receptor-positive. Tamoxifen reduced recurrence rates by approximately 68% (18). To date, this patient has not yet received endocrine therapy, but we believe the addition of endocrine therapy will provide this patient with longer survival benefit.

Breast cancer is the second most common malignancy causing brain metastases after lung cancer. Due to the presence of the blood-brain barrier, most chemotherapy drugs (such as taxanes, alkylating agents, and topoisomerase inhibitors) struggle to penetrate because of their large molecular weight and high polarity. Therefore, only by adding local therapy to brain metastases can long-term control of intracranial lesions and maximization of patient quality of life be achieved (19). Common local treatment approaches currently include local surgical intervention, whole-brain radiation therapy, stereotactic radiotherapy, and ablation (20). Surgical resection plays a crucial role in managing solitary lesions larger than 3 cm, those with mass effect, or those requiring definitive pathological diagnosis. Whole-brain radiotherapy is the standard treatment for multiple metastases. Stereotactic radiosurgery, as a high-precision, high-dose radiotherapy technique, can effectively control tumors while maximally preserving surrounding normal brain tissue (21, 22). Following four cycles of TX chemotherapy, the patient exhibited enlargement of the original brain metastases and the emergence of a new metastatic lesion. Consequently, we opted for whole-brain radiotherapy as the treatment modality. During radiotherapy, the patient did not experience common adverse effects such as cognitive decline. To date, no follow-up assessment of treatment efficacy has been conducted.

## 4 Conclusion

Isolated pulmonary lesions presenting as occult male breast cancer are extremely rare, and the limitations of imaging diagnosis can pose significant diagnostic challenges, making accurate diagnosis even more difficult. Accurate pathological diagnosis is the fundamental prerequisite for subsequent appropriate treatment and avoidance of ineffective or even harmful interventions. This case profoundly illustrates that when encountering male patients with imaging findings highly suggestive of primary lung cancer—especially when tumor markers or preliminary pathology results do not support a diagnosis of primary pulmonary tumor—clinicians must maintain high vigilance and include occult breast cancer within the differential diagnosis.

## Data Availability

The original contributions presented in the study are included in the article/**Supplementary Material**. Further inquiries can be directed to the corresponding author.
