# Supplementary figures and images for: Male occult breast cancer with features highly resembling primary lung cancer: a case report and literature review
Source: Front Oncol. 2026 Jan 12;15:1737310. doi: 10.3389/fonc.2025.1737310 (PMC12832376; doi:10.3389/fonc.2025.1737310)

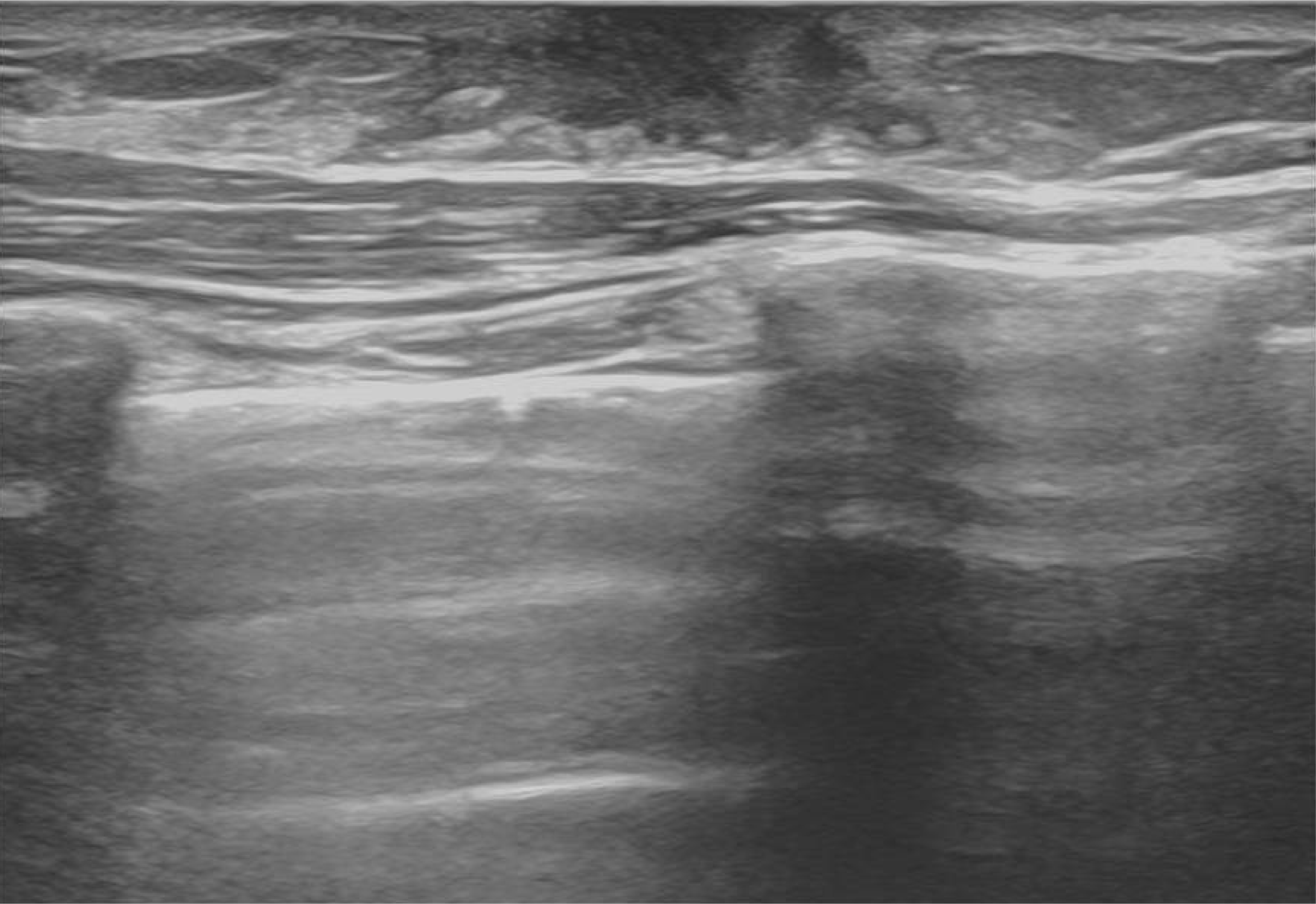

Supplement: Supplementary file 1 [file Image1.tif]

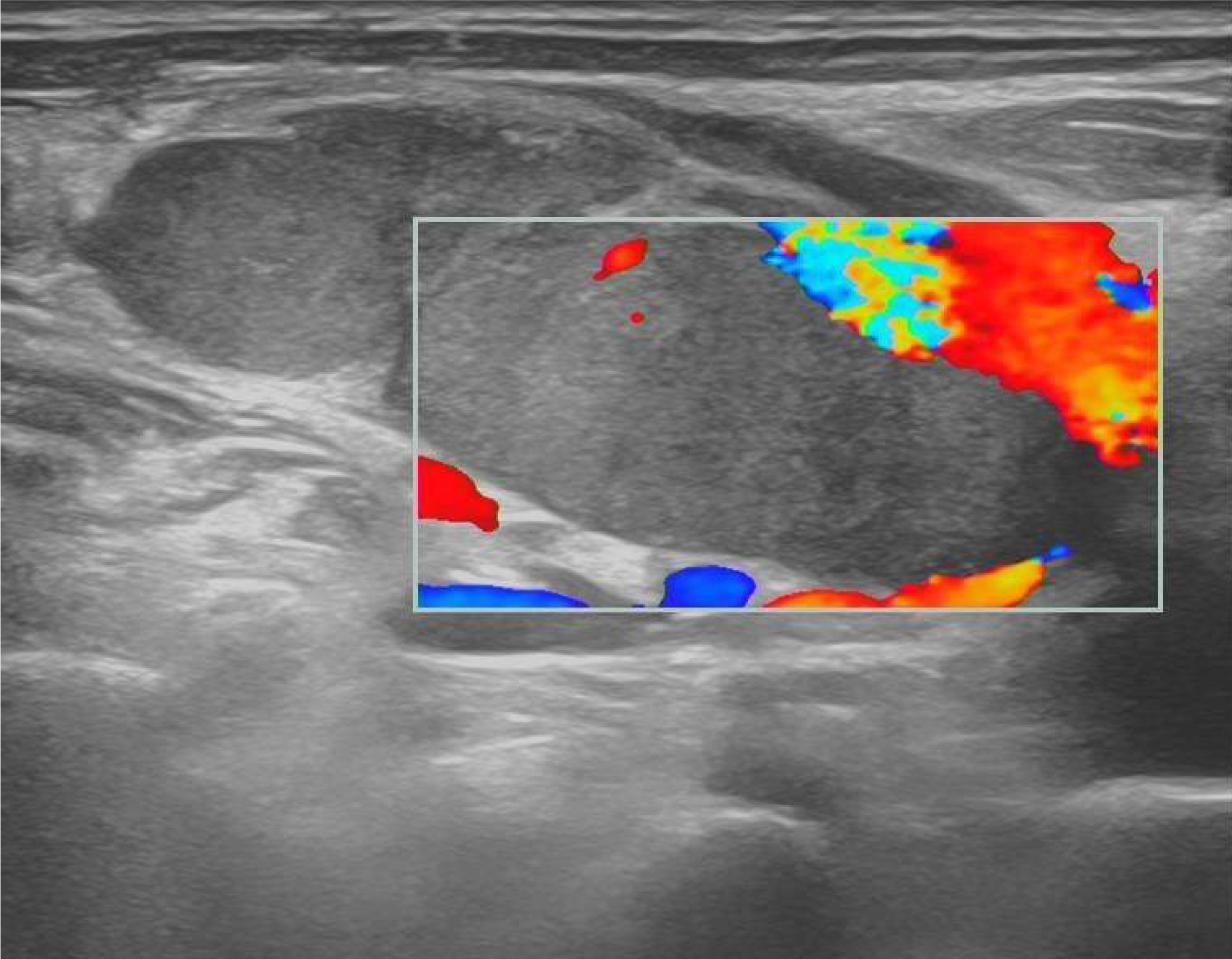

Supplement: Supplementary file 2 [file Image2.tif]

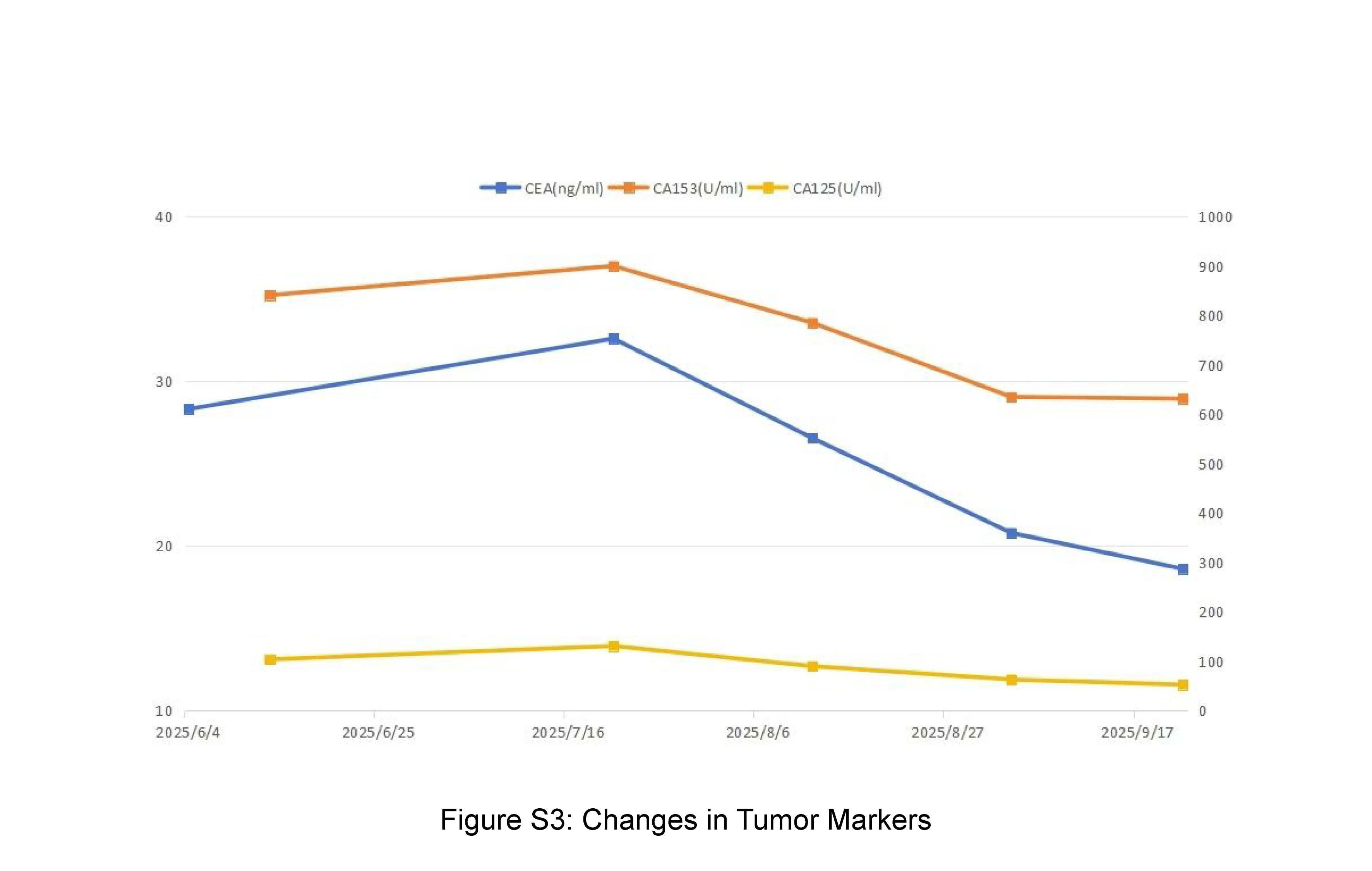

Supplement: Supplementary file 3 [file Image3.tif]
